# A Defined Synbiotic Produces Immunomodulatory Metabolites, Engages Gut–Immune Pathways Relevant to Inflammaging, and Supports Healthy Aging in a Nematode Model

**DOI:** 10.3390/ijms27146369

**Published:** 2026-07-17

**Authors:** Ryan S. Green, Daniela Diaz-Infante Morales, Eric M. Schott, Mark R. Charbonneau, Alicia E. Ballok

**Affiliations:** Sōlaria Biō, Waltham, MA 02453, USA; rgreen@solaria.bio (R.S.G.); eschott@solaria.bio (E.M.S.); mcharbonneau@solaria.bio (M.R.C.)

**Keywords:** probiotic, synbiotic, inflammaging, healthy aging

## Abstract

Chronic low-grade aging-associated inflammation, or inflammaging, is a central pillar of age-related decline in quality of life. Inflammaging is partially mediated by impaired intestinal, immune, and microbiome function, and it has been hypothesized that probiotics could be used to promote healthy aging. SBD121, a defined synbiotic containing food-derived microbial strains and prebiotic fibers, has previously been shown to improve grip strength in male rats, an important indicator of healthspan, and is under evaluation in a clinical trial of 143 newly diagnosed rheumatoid arthritis patients (NCT06005220). However, the mechanisms underlying its potential benefits have not been determined. Here, we examined the function of SBD121 in microbial, cellular, and animal models relevant to inflammaging. SBD121 inhibited the growth of potential microbial pathogens, produced immunomodulatory metabolites in vitro, and improved human intestinal cell barrier function under both basal and challenge conditions, while reducing inflammatory chemokine secretion following inflammatory challenge. SBD121 also reduced the secretion of multiple chemokines in lipopolysaccharide-stimulated human intestinal and immune cells. Finally, SBD121 improved survival and locomotor activity in a *C. elegans* longevity model, providing evidence of benefits to lifespan and healthspan. Together, these data demonstrate that SBD121 exhibits beneficial microbial, epithelial, immune, and longevity effects and support continued investigation of SBD121 as a candidate intervention for healthy aging.

## 1. Introduction

The global population aged 60 and older is projected to nearly double between 2015 and 2050, intensifying the public-health burden of age-associated chronic disease [1]. Chronic diseases of aging, including cardiovascular disease, type 2 diabetes, sarcopenia, and frailty, are associated with and exacerbated by low-grade aging-related inflammation, termed inflammaging [2,3,4,5,6,7]. Furthermore, inflammaging is increasingly recognized as a major contributor to age-related declines in quality of life [6,8].

Inflammaging is driven in large part by cellular senescence [4,9]. Briefly, accumulated insults, such as DNA damage and oxidative stress, activate two key senescence regulators, p21 (cyclin-dependent kinase inhibitor 1A; CDKN1A) and p16 (CDKN2A) [9,10]. These cell cycle inhibitors limit replicative potential and promote a senescence-associated secretory phenotype (SASP) [10,11]. This altered secretion profile is characterized by the secretion of inflammatory chemokines (C-C motif chemokine ligand 2 [CCL2], CCL20, interleukin-8 [IL-8], and C-X-C motif chemokine ligand 1 [CXCL1]) and cytokines (tumor necrosis factor a [TNF-α], IL-1β, and IL-6) [9,12]. These secreted factors result in maladaptive immune cell recruitment, chronic sterile inflammation, and induction of senescence phenotypes specific to immune cells (immunosenescence). Immunosenescence creates a paracrine signaling-driven feed-forward cycle in which SASP factors damage local tissue while recruiting and priming immune cells that further amplify tissue damage and immune cell activation, and induce bystander senescence [9]. Further, aging and immunosenescence shift adaptive immune responses toward T helper (Th) 17 responses, which are highly associated with chronic inflammatory disorders and autoimmune diseases [13,14,15]. In total, inflammaging and immunosenescence drive systemic tissue damage and impaired immune responses.

An increasingly recognized contributor to systemic inflammation, inflammaging, and immunosenescence is intestinal barrier dysfunction. The intestinal barrier normally functions to protect the body from microbial challenge and inappropriate immune responses directed towards gut commensal organisms [16]. With aging, the intestinal barrier exhibits increased permeability, due in part to broader systemic inflammation and to microbial dysbiosis [9,17]. The resulting “leaky gut” allows translocation of inflammatory microbial compounds which can activate the epithelium and surveilling immune cells, promoting inflammatory responses that can promote additional barrier dysfunction and immune cell recruitment. Further, these activated immune cells have the potential to circulate systemically and promote inflammation throughout the body [18,19].

Given the importance of the gastrointestinal (GI) tract to systemic inflammation and inflammaging, it has been hypothesized that probiotics and prebiotics have the potential to interrupt the inflammatory cycle of the gut–immune axis by enhancing GI barrier function [3,16,18]. Microbial metabolites, such as acetate and indole derivatives, enhance barrier function and can promote anti-inflammatory responses in immune cells [20,21,22,23,24]. Additionally, microbially produced phenolic antioxidants can mitigate tissue damage [25]. Finally, probiotics have been shown to produce neurotransmitters, such as gamma-aminobutyric acid (GABA) and serotonin, which influence signaling along the gut–brain axis, modulating homeostatic, stress, and emotional responses [26,27,28].

To this end, we have designed SBD121, a synbiotic composed of four fruit- and vegetable-derived bacterial strains (*Levilactobacillus brevis* strain SBS04254, *Bacillus amyloliquefaciens* strain SBS04877, *Schleiferilactobacillus harbinensis* strain SBS04913, and *Lactococcus lactis* strain SBS04916) and prebiotic fibers [29]. These microbial species were selected for their reported capacity to produce neuroactive, immunomodulatory, and antimicrobial compounds [26,30,31,32,33].

This synbiotic combination has been shown to be safe following oral administration in a 28-day toxicity study and to improve grip strength, a measure of healthspan, in male rats [29]. The safety profile of SBD121 is further supported by a randomized, open-label human clinical trial showing that it does not engraft in the microbiome [34]. SBD121 is also under investigation in a double-blind, randomized placebo controlled clinical trial measuring its effects on joint health in the context of patients recently diagnosed with rheumatoid arthritis (Clinicaltrials.gov number: NCT06005220). While SBD121 and its constituent microbes exhibit diverse effects in vivo, the specific mechanisms that enable SBD121 to impact microbial, epithelial, and immune responses relevant to inflammaging have not been determined.

Here, we tested the hypothesis that SBD121 modulates microbial, epithelial, and immune pathways implicated in inflammaging and healthy aging [35]. As SBD121 is administered orally via enterically coated capsules that release upon exposure to neutral pH in the ileum, its microbes and prebiotic fibers are protected from digestion and delivered directly to the small intestine [36]. Because of this targeted delivery, we first examined the ability of SBD121 and its individual constituent strains to inhibit potential microbial pathogens and to produce immunomodulatory and neuroactive compounds. We then examined its direct effects on host cells, first dissecting intestinal barrier responses to SBD121 and then evaluating immune cell responses. Finally, we examined the effects of SBD121 on lifespan and healthspan using a nematode aging model.

## 2. Results

### 2.1. SBD121 and Its Constituent Microbes Exhibit Anti-Pathogen Phenotypes and Produce Gut-Relevant Metabolites

Each constituent microbial species present in SBD121 has been reported to produce immunomodulatory and/or antimicrobial compounds relevant to the gastrointestinal tract [30,31,32,33]. Given the profound functional diversity seen within microbial species, we examined the ability of the SBD121 assemblage and each component strain to inhibit the growth of potentially pathogenic microbes and to produce compounds that could beneficially affect human cells.

**Figure 1:**
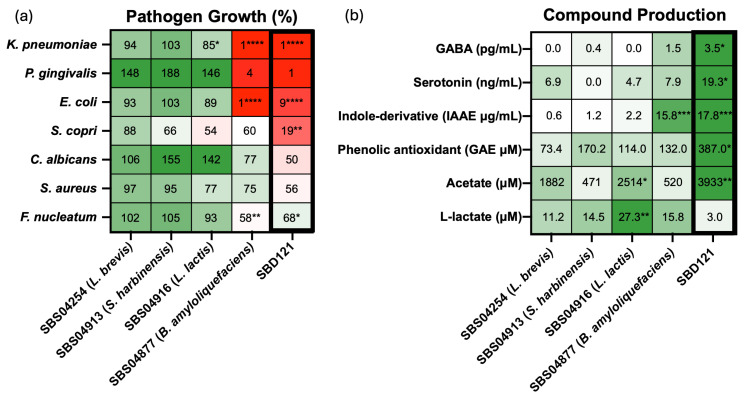
SBD121 inhibits pathogen growth and produces beneficial gut-relevant compounds. (**a**) SBD121 produces antimicrobial compounds. SBD121 or its constituent strains were grown aerobically for 48 h. After which the bacteria-free conditioned supernatants or a medium control were applied to potential pathogenic bacteria (*Klebsiella pneumoniae*, *Porphyromonas gingivalis*, *Escherichia coli*, *Segatella copri*, *Candida albicans*, *Staphylococcus aureus*, and *Fusobacterium nucleatum*). Color indicates degree of inhibition from not inhibited (green) to strongly inhibited (red). Data represent the mean of 2–3 independent experiments, each with 3–6 technical replicates per experiment. Significance was determined via One-way ANOVA with Dunnet’s HSD, comparing all conditions to SBD121 the media background. (**b**) SBD121 produces several immunomodulatory/neuroactive compounds. SBD121 or its component microbes was grown anaerobically for 48 h, after which gamma amino-butyric acid (GABA), serotonin, acetate, and L-lactate concentrations were determined. Alternatively, microbes were grown aerobically for 48 h to examine indole-derivative or phenolic antioxidant production. Color indicates the relative production of a metabolite from low (white) to high (green). Each data set corresponds to a representative experiment. IAAE refers to μg/mL indole-3-acetic acid equivalents while GAE μM refers to μM gallic acid equivalents. Significance for GABA and serotonin was determined via Welch ANOVA with Dunnet’s T3 multiple comparisons test comparing all conditions to the media background. Significance for indole derivatives, phenolic antioxidants, acetate, and L-lactate was determined using one-way ANOVA with Dunnet’s multiple comparisons test, comparing all conditions to the media background. * = *p* < 0.05, ** = *p* < 0.01, *** = *p* < 0.001, and **** = *p* < 0.0001.

As lactic acid bacteria and *Bacillus* spp. are well known to produce antimicrobial compounds, we first examined the ability of each SBD121 constituent strain and the total assemblage to inhibit the growth of potentially pathogenic microbes representing several taxa, including: *Klebsiella pneumoniae*, *Porphyromonas gingivalis*, *Escherichia coli*, *Segatella copri*, *Candida albicans*, *Staphylococcus aureus*, and *Fusobacterium nucleatum* [30,37,38,39,40,41]. Sterile-filtered supernatants from SBD121 capsule contents or from individual strains cultured in peptone yeast extract broth with glucose (PYG) for 48 h were applied to pathogen cultures, and anaerobic growth was assessed after 24, 48, or 72 h. Entire SBD121 capsule contents were utilized to model intestinal release of the complete synbiotic. Because prebiotics can serve as microbial nutrition sources that modulate SBD121 or pathogen viability, growth, or metabolite production, they were included in all studies examining the full synbiotic [42]. When pathogen growth was examined, *B. amyloliquefaciens* SBS04877 supernatants significantly inhibited *K. pneumoniae*, *E. coli*, and *F. nucleatum*, with nonsignificant inhibition observed against *P. gingivalis*, *S. copri*, *C. albicans*, and *S. aureus* (Figure 1a and Table S1). *L. lactis* SBS04916 supernatants significantly inhibited *K pneumoniae*, while *L. brevis* SBS04254 and *S. harbinensis* SBS04913 supernatants did not significantly inhibit any tested pathogen. Supernatants from the SBD121 combination exhibited similar inhibition phenotypes to *B. amyloliquefaciens* SBS04877 with significant inhibition of *K. pneumoniae*, *E. coli*, and *F. nucleatum*, and nonsignificant inhibition of *P. gingivalis*, *C. albicans*, and *S. aureus*. Notably, *B. amyloliquefaciens* SBS04877 maintained a near-neutral post-HEPES pH (7–7.5), yet demonstrated the strongest pathogen inhibition, supporting the conclusion that the observed inhibitory activity is attributable to strain-specific antimicrobial compounds rather than residual acidification. However, SBD121 demonstrated enhanced inhibition of *S. copri*, relative to its individual strains. These data indicate robust inhibition of diverse potentially pathogenic species.

The ability of SBD121 and its constituent microbes to produce gut-active neurotransmitters and immunomodulatory compounds was also evaluated (Figure 1b). SBD121 capsule contents or its component strains were incubated for 48 h, after which conditioned supernatants were analyzed for GABA, serotonin, indole derivatives, phenolic antioxidants, acetate, and L-lactate [43,44]. SBD121 produced significant concentrations of GABA, serotonin, indole derivatives, phenolic antioxidants, and acetate relative to the media background. By contrast, for individual component strains, significantly elevated concentrations of these metabolites relative to media controls were observed only for *B. amyloliquefaciens* SBS04877 (indole derivatives) and *L. lactis* SBS04916 (acetate, L-lactate). Notably, SBD121 did not produce significant amounts of L-lactate. These data suggest more robust production of neurotransmitters and immunomodulatory metabolites by the SBD121 constituent strains in combination than that observed for the strains in isolation. Furthermore, the SBD121 combination produced 3.5 µM GABA compared to a sum of 1.9 µM across all four individual strain means, representing approximately 1.8-fold greater production than the additive expectation. No individual strain achieved statistical significance relative to media, whereas the SBD121 combination was significant (*p* = 0.05).

### 2.2. SBD121 Enhances Intestinal Barrier Integrity While Reducing Human Intestinal Cell-Mediated Inflammatory Responses

Age-associated intestinal barrier dysfunction contributes to inflammaging by enabling translocation of luminal microbial products that trigger immune activation, immune cell recruitment, and sustained tissue damage [16,18]. Given that SBD121 was observed to produce several relevant immunomodulatory compounds, we examined its ability to regulate intestinal barrier integrity and immune cell recruitment mediated by chemokine secretion from human intestinal epithelial cells (IECs).

**Figure 2:**
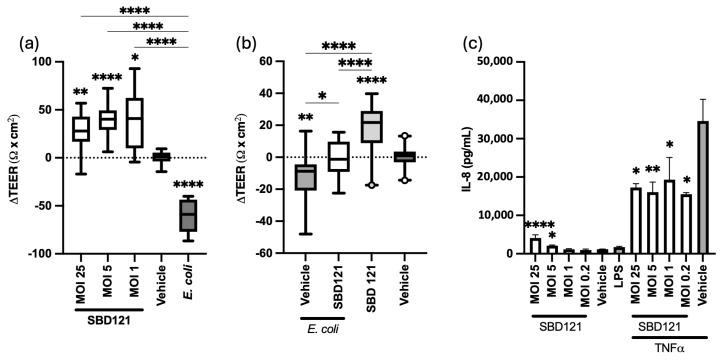
SBD121 enhances intestinal epithelial cell barrier function (transepithelial electrical resistance [TEER]) while reducing inflammatory signaling in the context of inflammatory challenge. (**a**) Mature, polarized intestinal epithelial cell (IEC) monolayers (Caco-2 and HT29 cells) were treated with SBD121 capsule material (Multiplicity of interaction [MOI] 25, 5 or 1), a media control (vehicle) or an *E. coli* disruption control. TEER was measured before and 16 h after administration and the mean change in TEER (ΔTEER) was compared across different conditions. Data presented is a combination of two experiments (N = 12–16 per condition). The central line within each box represents the mean of each condition, with boxes representing interquartile range (IQR) and error bars indicating the minimum and maximum values. Significance between conditions was determined by Welch ANOVA with Dunnett’s T3 multiple comparisons test. (**b**) Mature, polarized IEC monolayers were pretreated with *E. coli* or media for 4 h. Subsequently, monolayers were treated with polymyxin B (an *E. coli* specific antibiotic) and SBD121 capsule material (MOI 25, 5 or 1) or a media control (vehicle). ΔTEER was determined after 16 h. Presented data represents three combined experiments, N = 15–20 per condition. (**c**) Non-polarized HT29 IEC monolayers were co-treated with TNF-*α* and SBD121 (MOI 25, 5, 1, or 0.2) or a vehicle control, or a stimulatory control (lipopolysaccharide, LPS). After 24 h supernatants were harvested and IL-8 concentrations were determined via ELISA. Data presented is a single representative of three experiments. For (**a**) and (**b**) central line within each box represents the mean of each condition, with boxes representing interquartile range (IQR) and error bars indicating the minimum and maximum values. For panel (**c**) bars indicate the mean of the condition ± the standard deviation (SD). Significance was determined by one-way ANOVA with Tukey’s HSD. Asterisks lacking comparison bars identify significance relative to the vehicle control. * = *p* < 0.05, ** = *p* < 0.01, **** = *p* < 0.0001.

To assess effects on barrier integrity, baseline transepithelial electrical resistance (TEER) across human polarized IEC monolayers was determined. Cells were apically treated with a media control (vehicle), *E. coli* (a microbe known to disrupt epithelial barriers), or SBD121 capsule contents, at a multiplicity of interaction (MOI; microbes per human cell) of 25, 5, or 1 [45]. These MOIs were chosen to represent a range of low to very high local concentrations that could be observed in someone taking SBD121. Based on a human SBD121 dose of 5.2 × 10^10^ CFU, an estimated ileal surface area of ~18 m^2^, and intestinal epithelial cell densities of 4.2 × 10^6^ to 1.9 × 10^7^ cells/cm^2^ (derived from human colonic biopsy and murine jejunal analyses), IECs are expected to experience an SBD121 exposure of approximately MOI 1.5–6.9 [46,47,48,49]. Following a 16 h incubation with each treatment, the change in TEER from baseline (ΔTEER) was determined as a measure of barrier permeability and function. SBD121 improved TEER relative to a vehicle control across a broad concentration range (Figure 2a). *E. coli* reduced TEER as expected. SBD121 did not induce polarized IEC monolayers to produce IL-8 or CXCL1, inflammatory chemokines associated with neutrophil recruitment, at any concentration (Figure S1a,b).

Given that aging is associated with barrier disruption and intestinal dysbiosis, we next determined whether SBD121 would benefit polarized IEC monolayers pretreated with *E. coli*, a pathogen whose overgrowth is commonly associated with intestinal dysbiosis and barrier dysfunction [17,45]. After pretreatment, each monolayer was treated with polymyxin B, a Gram-negative specific antibiotic, and SBD121 (MOI 5, the concentration that provided the most significant improvement during challenge-free conditions) or a media control (vehicle). After 16 h, ΔTEER was again determined. SBD121 improved TEER relative to the vehicle control, while *E. coli* reduced TEER (Figure 2b). Importantly, SBD121 also significantly mitigated *E. coli*-induced barrier damage, restoring TEER to unchallenged levels. These data indicate that SBD121 not only improves intestinal barrier function in intact IEC monolayers but can also restore intestinal barrier integrity in the context of pathogen disruption.

Beyond its role as a physical barrier, the intestinal epithelium also functions as an immunological regulator, detecting microbe-associated molecular patterns and responding by secreting chemokines and cytokines to recruit and prime immune cell reinforcements [16,17,18]. In the context of inflammaging, these reinforcements can become deleterious, resulting in local tissue damage and further barrier dysfunction [9,17,18]. Because the intestinal epithelium contributes to immune regulation, we next determined whether SBD121 affected IEC chemokine secretion under basal conditions and during inflammatory challenge. Unpolarized cells were treated with TNF-α or a media control (vehicle). TNF-α was selected for this model because IECs commonly interact with microbes and are less responsive to microbially derived stimuli, as reflected by comparison between the LPS control and TNF-α controls in Figure 2c [50]. Concurrently, these monolayers were co-treated with a range of SBD121 concentrations (MOI 25, 5, 1, or 0.2), a vehicle control, or lipopolysaccharide (LPS; a microbe-derived inflammatory control). Cells were incubated for 24 h and chemokine secretion was measured (Figure 2c). We observed that in the absence of inflammatory challenge, SBD121 dose-dependently increased IL-8 (Figure 2c) and CXCL1 (Figure S1c) secretion by unpolarized IECs. In contrast, in the presence of an inflammatory challenge, SBD121 reduced IL-8 and CXCL1 secretion at all tested concentrations. These findings suggest that SBD121 can modulate epithelial immune signaling in a context-dependent manner, increasing chemokine secretion under basal conditions while limiting inflammatory chemokine secretion during TNF-α challenge.

### 2.3. SBD121 Limits Immune Cell Recruitment Signals from PBMCs During Inflammatory Challenge

As aging is associated with intestinal barrier disruption and the translocation of inflammatory microbes and microbial products into the lamina propria, activation of underlying immune cells is a major driver of inflammaging [9,17]. GI-resident immune cells interact with microbes and their products by transepithelial sampling, microfold cell-mediated translocation, and barrier dysfunction [51,52]. Further, these immune cells are known to circulate systemically and elicit effects throughout the body [19]. For these reasons we next determined how SBD121 affects immune cell responses.

**Figure 3:**
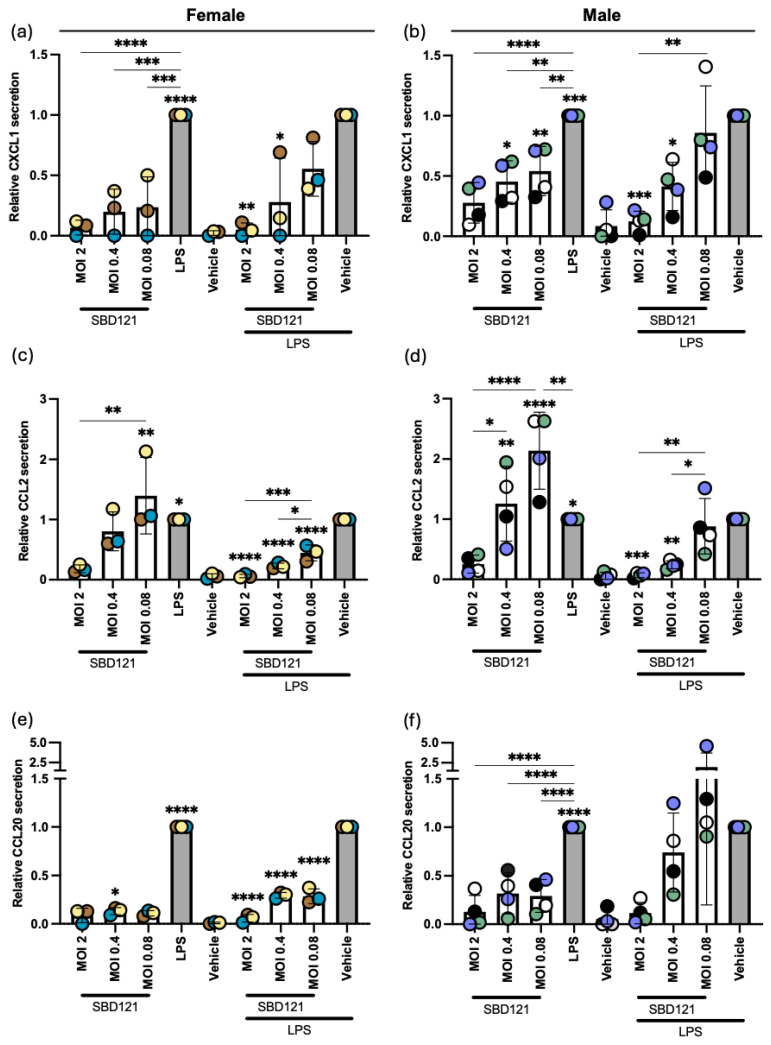
SBD121 administration reduces chemokine secretion by PBMCs after inflammatory challenge. Human PBMCs from seven healthy donors (three female (**a**,**c**,**e**) and four male (**b**,**d**,**f**)) were pretreated with media or LPS (100 ng/mL) to induce an inflammatory response. Subsequently cells were treated with SBD121 material (MOI 2, 0.4, or 0.08), a stimulatory control (LPS) or a media control (Vehicle). After 16 h chemokine secretion was determined via ELISA (CXCL1 (**a**,**b**), CCL2 (**c**,**d**), and CCL20 (**e**,**f**)). To compare data across donors, inflammation naïve and LPS challenge conditions were normalized to LPS and LPS-challenged, vehicle-treated controls, respectively. Columns are indicative of the mean ± SD for all the donors of an individual sex. Points indicate individual donors and are color coded by donor. Females: Donor 1 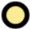; Donor 2 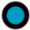; Donor 3 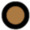. Males: Donor 4 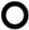; Donor 5 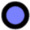; Donor 6 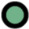; Donor 7 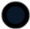). Significance was determined by One-way ANOVA with Tukey’s HSD. Asterisks without comparison bars indicate significance relative to the vehicle control. * = *p* < 0.05, ** = *p* < 0.01, *** = *p* < 0.001, **** = *p* < 0.0001.

**Figure 4:**
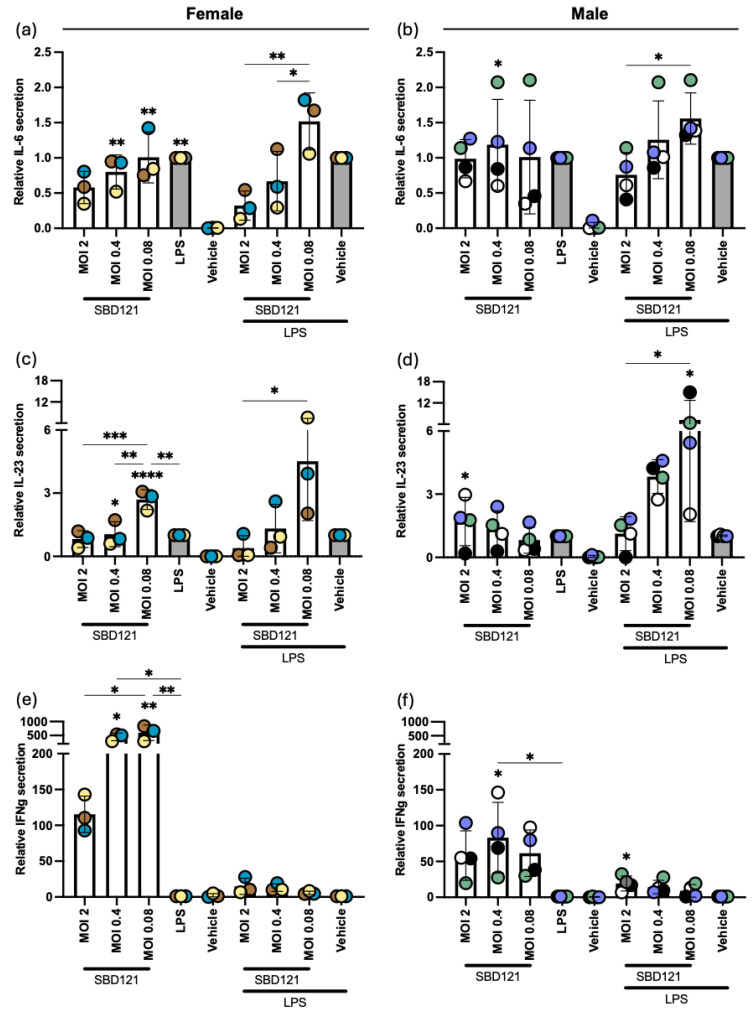
SBD121 administration dosed-dependently promotes Th1 cytokine secretion by PBMCs at baseline and after inflammatory challenge. Healthy donor PBMCs (three female (**a**,**c**,**e**) and four male (**b**,**d**,**f**)) were pretreated with media or LPS (100 ng/mL), inducing an inflammatory response. After which cells received SBD121 capsule contents (MOI 2, 0.4, or 0.08), a stimulatory control (LPS) or a media control (Vehicle). T cell polarizing cytokine secretion was determined via ELISA after 16 h (IL-6 (**a**,**b**), IL-23 (**c**,**d**), and IFN-*γ* (**e**,**f**)). Multiple donors were compared by normalizing inflammation naïve and LPS challenge conditions to LPS and LPS-challenged, vehicle-treated controls, respectively. Columns indicate the mean ± SD for all the donors of an individual sex. Points indicate individual donors and are color coded by donor. Females: Donor 1 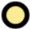; Donor 2 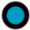; Donor 3 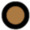. Males: Donor 4 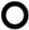; Donor 5 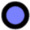; Donor 6 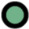; Donor 7 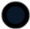). Significance was determined by One-way ANOVA with Tukey’s HSD. Asterisks without comparison bars indicate significance relative to the vehicle control. * = *p* < 0.05, ** = *p* < 0.01, *** = *p* < 0.001, **** = *p* < 0.0001.

To determine the impact of SBD121 on immune cell responses, we used human peripheral blood mononuclear cells (PBMCs), which are commonly used to model probiotic–immune cell interactions [53,54,55]. PBMCs isolated from seven healthy donors (three female, four male) were pretreated with an inflammatory stimulus (LPS) or media alone, then exposed to SBD121 (MOI 2, 0.4, or 0.08), a media control (vehicle), or a stimulatory control (LPS, challenge-naïve condition only). These SBD121 concentrations were used to model the microbial concentrations that PBMCs would experience in vivo. Unlike IECs, which are in direct contact with luminal contents, PBMCs in the lamina propria and circulation are separated from the intestinal lumen by the epithelial barrier and would encounter far fewer microbes under homeostatic conditions. The higher MOI (2) was included to model concentrations that could be experienced during barrier dysfunction, consistent with the previously determined physiological IEC range (MOI 1.5–6.9). Given the pronounced effect of SBD121 on IEC chemokine responses (Figure 2c), we first examined how SBD121 altered PBMC chemokine secretion (Figure 3 and Figure 4a,b). In the absence of inflammatory challenge, low SBD121 concentrations resulted in increased secretion of CXCL1, CCL2, and CCL20 relative to the vehicle control. At high SBD121 concentrations (MOI 2), chemokine secretion returned to levels that were not significantly different from the vehicle control. This trend was conserved across both sexes but was not significant for male CCL20 secretion. Given the small number of donors per sex (*n* = 3 female, *n* = 4 male), all sex-stratified comparisons are presented as preliminary. The same trend was seen for the neutrophil-attracting chemokine IL-8 during inflammatory challenge but not under basal conditions (Figure S1a,b). In the context of inflammatory challenge, SBD121 reduced CXCL1 and CCL2 secretion below the vehicle control across both sexes (Figure 3a–d). CCL20 exhibited a similar trend but was only significant for PMBCs from female donors (Figure 3e,f).

### 2.4. SBD121 Modulates Th17- and Th1-Associated Cytokine Responses in Human Immune Cells

Given that T helper cell polarization broadly shapes immune responses, we next examined how SBD121 affects the cytokines that drive and maintain disctinct T helper cell states (Figure 4). Specifically, Th17 responses are linked with inflammaging and autoimmune disease, so we initially examined the Th17-associated cytokines, IL-6 and IL-23 [13,14]. Cytokine responses to SBD121 showed sex-dependent patterns under challenge-naive conditions but converged under inflammatory challenge. Specifically, in the absence of inflammatory challenge, sex-based differences were seen, with females exhibiting cytokine induction at low MOIs that trended downward as MOI increased (Figure 4a,c). Males exhibited elevated IL-6 and IL-23 secretion that remained elevated as SBD121 concentration increased, which was significant for IL-23 (Figure 4b,d). However, in the context of inflammatory challenge, IL-6 and IL-23 did not exhibit sex-based differences: low SBD121 concentrations resulted in nonsignificantly increased IL-6 and IL-23 secretion, which was significantly reduced in a concentration-dependent manner, relative to low MOI treatment (Figure 4a–d).

We next examined the Th1-associated cytokine, interferon-gamma (IFN-γ) [56]. Th1 responses represent inflammatory responses that are associated with greater resolution than Th17 responses and can mitigate tissue damage from intestinal helminth challenges [15,57]. In challenge-naïve PBMCs, IFN-γ secretion increased following SBD121 administration, with females exhibiting a significant reduction in IFN-γ secretion as SBD121 concentration increased (Figure 4e,f). During inflammatory challenge, responses followed a different pattern, characterized by concentration-dependent increases in IFN-γ secretion but lower total secretion relative to the challenge-naïve condition. This shifting Th1/Th17 profile was further confirmed by examination of an additional Th1 polarizing cytokine (IL-12) and Th17 effector cytokine (granulocyte-macrophage colony-stimulating factor, GM-CSF) which trended similarly to IFN-γ and IL-23 secretion patterns, respectively (Figure S2) [56].

Finally, we examined other SASP cytokines, TNF-α and IL-1β. Both cytokines exhibited robustly increased secretion in response to SBD121 under basal and inflammatory conditions, although not significantly for males under challenge (Figure S3). This increase in secretion is not unexpected as these are primary inflammatory cytokines secreted in response to bacteria [54,58]. Additionally, we examined anti-inflammatory cytokines to determine if SBD121 exerted its influence through direct anti-inflammatory responses [54,58]. Similar to the examined chemokines, these anti-inflammatory cytokines (IL-10 and IL-1RA) were induced by low concentrations of SBD121 and exhibited dose-dependent decreases in secretion as SBD121 concentrations increased, at baseline and challenge conditions (Figure S4). Together, these data suggest that SBD121 induces T cell responses in a context- and sex-dependent manner. Under inflammatory challenge, low SBD121 concentrations were associated with greater Th17-related cytokine secretion, whereas higher SBD121 concentrations were associated with reduced IL-6 and IL-23 and increased IFN-γ secretion.

### 2.5. SBD121 Reduces Expression of a Senescence Marker in Human Immune Cells During Inflammatory Challenge

One characteristic of inflammaging is the induction of senescence. Senescence is a unique cellular state wherein immune and other cell types cease replicating and begin to exhibit a dysfunctional inflammatory phenotype described as the SASP [9,10]. This shift in cellular secretion further drives much of the systemic inflammation and tissue damage characteristic of inflammaging [2,9]. The induction of senescence is primarily mediated by the cyclin-dependent kinases p21 and p16, with p21 being the primary driver of acute cellular senescence [12]. Given that SBD121 downregulates several conserved SASP proteins in PBMCs (CXCL1, IL-8, CCL2, and CCL20), we next examined whether this observation was associated with changes in p21 gene expression [9,10,11,12].

**Figure 5:**
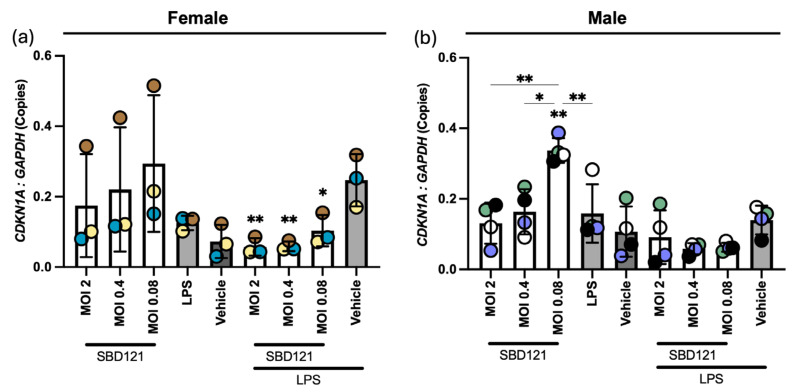
SBD121 administration reduced senescence marker expression by PBMC after inflammatory challenge. Healthy donor PBMCs (three female (**a**) and four male (**b**)) were pretreated with media or LPS (100 ng/mL), to produce an inflammatory response. After which cells received SBD121 capsule contents (MOI 2, 0.4, or 0.08), a stimulatory control (LPS) or a media control (Vehicle) for 16 h. Gene expression of the senescence marker CDKN1A (p21) was determined by qRT-PCR. Copy numbers were extrapolated using a standard curve, and data is presented as the copies of CDKN1A per copy of GAPDH. Columns indicate the mean +/− standard deviation for all the donors of an individual sex. Points indicate individual donors and are color coded by donor. Females: Donor 1 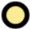; Donor 2 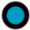; Donor 3 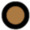. Males: Donor 4 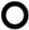; Donor 5 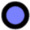; Donor 6 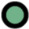; Donor 7 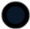). Significance was determined by One-way ANOVA with Tukey’s HSD. Asterisks without comparison bars indicate significance relative to the vehicle control. * = *p* < 0.05, ** = *p* < 0.01.

RNA was harvested from the PBMC experiments described previously, and total abundances of p21 transcripts were determined via quantitative reverse transcriptase polymerase chain reaction (qRT-PCR). In challenge-naïve PBMCs, SBD121 induced p21 expression at low MOIs which fell to media control levels as concentrations increased (Figure 5). This response was significant for males and trended similarly for females. During inflammatory challenge, both sexes exhibited trends of reduced p21 expression at low SBD121 concentrations, with females maintaining significantly reduced p21 expression relative to the challenged vehicle control at all tested concentrations. Expression of p16, a senescence marker associated with chronic challenge, was also examined but was not significantly affected by SBD121 administration (Figure S5) [11].

### 2.6. SBD121 Increases Lifespan and Healthspan in a Nematode Model of Aging

**Figure 6:**
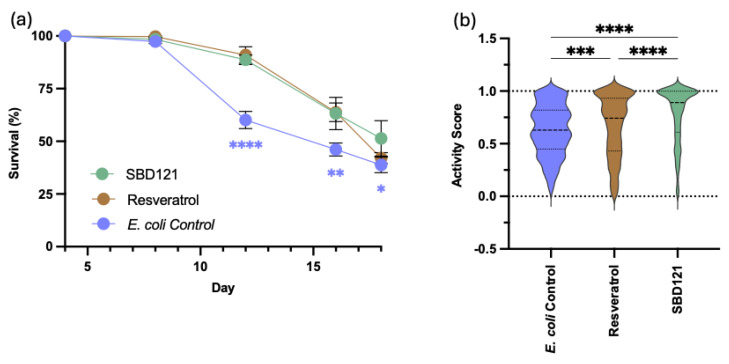
SBD121 enhances lifespan and activity in a *C. elegans* model of aging. *C. elegans* was grown on NGM supplemented with SBD121 synbiotic, *E. coli* (OP50), or a resveratrol control. (**a**) Percent survival was determined on days 4, 8, 12, 16, and 18. Data represents the combined result of two separate experiments. Statistical significance was determined via mixed-effects analysis with Geisser-Greenhouse correction and Dunnett’s multiple comparison test comparing all conditions to SBD121. Asterisk coloration indicates the compared condition. (**b**) On day 16 nematode activity levels were determined through video documentation of nematode movements. Violin plot width represents the percentage of nematodes exhibiting each activity score. Central dotted lines within the plot represents the median activity score, and the peripheral dotted lines within each plot represent the IQR. Statistical significance was determined via Kruskal-Wallis test with Dunn’s multiple comparisons test. * = *p* < 0.05, ** = *p* < 0.01, *** = *p* < 0.001, **** = *p* < 0.0001.

Given the observed effects of SBD121 on modulating markers associated with gut health, inflammatory responses, and immunosenescence, we examined the effects of SBD121 administration in a *Caenorhabditis elegans* model of aging. As aging is a complex, organism-level phenomenon, nematode models have been developed to examine diverse effects on lifespan and healthspan [35]. In this model, *C. elegans* strain N2 nematodes were maintained on nematode growth medium (NGM) with *E. coli* OP50 as the standard food source. The animals were exposed to resveratrol in the presence of OP50 as a longevity control, OP50 alone as a negative control, or SBD121 synbiotic as the test condition. Nematode survival was measured over 18 days and nematode locomotor activity was determined on day 16. We observed that SBD121 improved survival similar to the resveratrol positive control, with survival improvements seen at all timepoints after day 8 (Figure 6a). Furthermore, SBD121 significantly increased aged nematode locomotor activity relative to both resveratrol and the *E. coli* controls (Figure 6b). Together these data indicate that SBD121 administration improved lifespan to an extent comparable to resveratrol while having a greater effect on healthspan than resveratrol.

## 3. Discussion

An increasingly aged population and rising incidence of chronic diseases of aging has led to healthy aging being recognized as a global health concern, where necessary interventions and support are lacking [1,6,7,8]. To this end, we have developed SBD121, a synbiotic combination of four probiotic strains and prebiotic fibers, designed based on the potential of its individual bacteria to produce immunomodulatory, neuroactive, and antimicrobial compounds with potential relevance to healthy aging [20,26,31,32]. Herein, we further interrogated the individual mechanisms through which SBD121 may influence healthy aging, including antimicrobial effects, the production of gut-relevant compounds, effects on intestinal barrier function, influences on human immune cells, and impacts on lifespan and healthspan in a *C. elegans* model. Critically, while the individual bacterial species within SBD121 have documented functions, we demonstrate that their symbiotic combination produces emergent properties not observed in individual strains alone, including enhanced *S. copri* inhibition, neurotransmitter production, and antioxidant activity, as well as evidence of cross-feeding interactions, supporting the novelty of this defined synbiotic formulation. This is further exemplified by GABA production, where the SBD121 combination produced approximately 1.8-fold more GABA than predicted by summing the individual strain means, consistent with emergent metabolic activity from the combination.

First, we described the ability of SBD121 to inhibit numerous inflammatory and potentially pathogenic gut microbes. Each potentially pathogenic microbe tested was selected for its association with gastrointestinal dysbiosis (*E. coli*, *K. pneumoniae*, *S. copri*), promotion of systemic inflammation (*S. copri*, *F. nucleatum*, *P. gingivalis*), or the ability to cause disease (*S. aureus*, *E. coli*, *K. pneumoniae*, *C. albicans*) [38,39,40,41]. This result is expected as *Lactococcus lactis* strains have been documented to produce antimicrobial peptides and *Bacillus amyloliquefaciens* is known to produce several antimicrobial compounds, including surfactin and fengycin [31,33]. It is important to note that microbial growth in vitro can be inhibited by media acidification, which is controlled in this assay by buffering of the SBD121 conditioned media. SBD121 exhibited similar pathogen growth inhibition to *B. amyloliquefaciens* SBS04877 alone for most pathogens tested, indicating that the probiotic combination does not have deleterious effects on the antimicrobial properties of *B. amyloliquefaciens* SBS04877. Given the significant inhibition of several pathogens and the trending inhibition of all tested pathogens, these data suggest that benefits associated with SBD121 administration may be due in part to inhibition of inflammatory enteric microbial species.

Probiotics are also known to produce numerous compounds that can influence microbiome composition, epithelial barrier function, immune responses, and the gut–brain axis [3,42]. As such, we examined the ability of SBD121 and its constituent microbes to produce a selection of gut-relevant compounds thought to promote healthy aging. We found that SBD121 produced significant amounts of the neurotransmitters GABA and serotonin, molecules that play diverse roles in the gut. GABA has been shown to influence the gut–brain axis through the vagus nerve and promote anti-inflammatory responses in immune cells, while gastrointestinal serotonin has been shown to support peristalsis and modulate immune responses, processes that are all negatively impacted during aging [26,27,28]. Additionally, we showed that SBD121 produces other immunomodulatory compounds, including phenolic antioxidants, indole-3-acetic-acid (IAA)-like metabolites, and acetate. Phenolic antioxidants reduce oxidative stress, relieving a source of tissue damage and senescence induction [25]. Acetate and indole derivatives, such as IAA, enhance the intestinal epithelial barrier while promoting anti-inflammatory responses through regulation of Th17/Treg balance (indole derivatives), anti-inflammatory B10 cells (acetate), and microbial cross-feeding, supporting butyrate production by commensal microbes [20,21,23,24]. When individual microbes were examined, *B. amyloliquefaciens* 04877 was observed to produce significant levels of indole derivatives, while *L. lactis* SBS04916 produced significant levels of acetate and L-lactate. SBD121 also robustly produced indole derivatives and acetate but did not produce L-lactate. The loss of L-lactate production by SBD121, compared to *L. lactis* SBS04916, suggests that L-lactate may be consumed via cross-feeding by other members of the SBD121 consortium [59]. Phenolic antioxidants were significantly produced by the SBD121 combination, while the individual microbes did not. These data support our hypothesis that SBD121 incorporates, and in some cases enhances, the phenotypes of its constituent microbial strains, which could promote healthy aging through complementary mechanisms. In future studies, it would be beneficial to determine if this putative cross-feeding relationship supports the growth of other members of SBD121 or the entire combination [34].

Given that SBD121 was demonstrated to produce intestinal epithelium-enhancing metabolites, we next investigated whether this synbiotic could support intestinal barrier function [9,17,20]. We observed that SBD121 improved barrier function in mature polarized monolayers while also ameliorating *E. coli*-mediated barrier disruption. In addition to their barrier functions, IECs likely represent the first cells that may induce an immune response after encountering SBD121 [16,50]. As such, we examined the chemokines produced by SBD121-stimulated unpolarized monolayers in the absence and presence of an inflammatory cytokine challenge (TNF-α). We observed that SBD121 induced moderate chemokine secretion under naïve conditions but inhibited inflammatory responses following strong inflammatory challenge. These data indicate that SBD121 may reduce the inflammatory response associated with inflammaging and other chronic inflammatory challenges, while maintaining the ability of the intestinal barrier to recruit additional immune cells during microbial challenges. Given that microbial translocation into the lamina propria, caused by “leaky gut”, is a major driver of inflammaging, mitigation of inflammatory responses alongside the improvement to barrier function could synergize to benefit healthy aging under homeostatic and inflammatory circumstances [16,17,18]. Furthermore, as microbial dysbiosis is also common during aging, these improvements to the intestinal epithelium could be enhanced by SBD121-mediated pathogen inhibition to further improve GI health [13,17,39]. It should be noted that these experiments utilized immortalized cell lines (Caco-2 and HT29), and thus may not fully recapitulate the barrier properties or inflammatory responses of primary or aged intestinal epithelium; future studies using primary intestinal organoids or aged epithelial models would strengthen these findings.

The gut represents an important immunological organ. Immune cells therein surveil the gut contents, protect against microbial overgrowth, and circulate systemically to elicit distal responses [16,52]. Given the importance of these cells to both inflammaging and immunosenescence, we examined the effect of SBD121 on human immune cells [2,9]. We found that SBD121 elicited secretion of several SASP chemokines by PBMCs under challenge-naïve conditions, including CXCL1, CCL2, and CCL20. Chemokine secretion under unchallenged conditions was reduced as SBD121 concentration increased, becoming not significantly different from the vehicle control. In contrast, SBD121 administration robustly inhibited chemokine secretion by PBMCs during inflammatory challenge. As these chemokines attract a range of cell types, neutrophils (CXCL1, IL-8), monocytes/macrophages (CCL2), and T cells (CCL2, Th1 cells; CCL20, Th17 cells), this phenotype represents a major disruption to cellular recruitment [60]. Alongside the inhibition of IEC chemokine secretion, SBD121 may robustly inhibit immune cell recruitment during inflammatory challenges, a phenotype that may intercept the feed-forward cycle of inflammation, tissue damage, and immune recruitment that drives barrier disruption [9,17,19]. Future studies could expand these findings by examining these responses in vivo, potentially by examining serum LPS/zonulin and intestinal immunophenotyping following SBD121 administration in the context of inflammatory challenge [61].

Interestingly, other PBMC-secreted inflammatory cytokines exhibit different response patterns compared to chemokine responses. Innate immune response cytokines (TNF-α and IL-1β) exhibit elevated secretion across all SBD121 concentrations and challenge conditions. In addition, Th17-polarizing/maintaining cytokines (IL-6 and IL-23) exhibit sex-specific responses during challenge-naïve conditions, which may be attributable to the limited number of donors that were analyzed [56]. In contrast, during inflammatory challenge, male and female Th17 responses (IL-6, IL-23, and GM-CSF) aligned, with SBD121 inducing cytokine secretion at low MOI but decreasing cytokine secretion at higher MOI in a concentration-dependent manner. Conversely, Th1-associated cytokines (IFN-γ and IL-12) exhibited robust induction during the challenge-naïve condition, with a trend toward elevation during the inflammatory challenge [56]. This interpretation is supported by the innate inflammatory and anti-inflammatory cytokines examined. SBD121 induced TNF-α and IL-1β, consistent with activation of innate immune signaling, while low SBD121 concentrations also induced IL-10 and IL-1RA, suggesting a coordinated anti-inflammatory counter-regulatory response [62]. In total these responses demonstrate that SBD121 modulates immune responses in a context-dependent fashion that may modulate pathways associated with inflammaging without inducing immunosuppression. These data indicate a potential shift away from Th17 responses toward Th1 responses, which may offer benefits to healthy aging since Th17 responses become overrepresented during aging and can result in chronic inflammation and autoimmunity [13,14,15]. Meanwhile, robust Th1 responses may provide benefit by enhancing phagocytosis, which can become dysfunctional during aging, and resolving more readily than Th17 responses [9,15,53]. Additionally, Th1 responses have been shown to mitigate tissue damage during dysfunctional Th17 and Th2 responses [15,57]. This response could function with reductions in chemokine secretion, as described above, to break the inflammatory cycle. The mechanism underlying this context-dependent response remains to be determined. Several hypotheses warrant investigation, including direct competition for pattern recognition receptor engagement, priming of resting immune cells via trained immunity mechanisms, or metabolite-mediated activation of anti-inflammatory pathways such as GPR41/43 or AhR signaling [20,23,63]. Future studies employing pathway inhibitors or neutralizing antibodies will be needed to distinguish between these possibilities. A limitation of the current PBMC studies is that donors ranged in age from 33 to 41 years and were exposed to SBD121 under acute conditions, which may not fully reflect the immune environment of an aging population under chronic probiotic supplementation; future studies should incorporate donors over 50 years of age and chronic dosing models to better characterize age-dependent immune responses to SBD121.

Given that several SASP-associated chemokines were impacted by SBD121 administration, we next sought to determine whether markers associated with immunosenescence were affected. P21 is a cell cycle inhibitor that can be activated by acute stress and represents a characteristic marker of immunosenescence [9,10,11]. Upon examination of p21 (*CDKN1A*) gene expression, we found that p21 mirrored the pattern of chemokine secretion. Under low-inflammatory conditions (challenge-naïve), low concentrations of SBD121 induced p21 expression, whereas p21 expression decreased as SBD121 concentration increased. Under inflammatory conditions, such as those that present as a function of aging, p21 expression decreased below the control for females and trended similarly for males following SBD121 administration. During inflammatory challenge male responses diverged at high SBD121 concentrations (MOI 2), with some males exhibiting a trending increase in p21 expression. As sex is documented to affect senescence, further research will examine additional donors to determine if this difference in SBD121 response is sex- or donor-dependent [4]. In contrast, p16, a marker associated with established chronic senescence, was not significantly affected by SBD121 administration. This is consistent with the acute nature of the experimental design, as p16 upregulation is associated with long-term replicative senescence rather than acute stress responses [10]. Future studies examining SBD121’s effects on chronically stressed or aged immune cells would be better suited to detect changes in p16 expression.

Finally, we evaluated whether the microbial, epithelial, and immune phenotypes observed in vitro were accompanied by organismal benefits in a *C. elegans* aging model [35]. Administration of SBD121 synbiotic extended lifespan to a degree comparable to the antioxidant resveratrol, while further enhancing healthspan, measured as nematode locomotor activity, above that of resveratrol-treated worms. These data are consistent with the improved grip strength that was observed previously in male rats following SBD121 administration and demonstrate the multifaceted potential of this synbiotic [29].

While these observations are encouraging, this model has notable limitations. In particular, this model is highly sensitive to nematode nutrition. As such, these lifespan improvements could be influenced by antioxidant content in SBD121, osmotic effect from the prebiotic, or by nutritional differences between SBD121 and the control *E. coli* OP50. Without a prebiotic-only control condition, these observations therefore cannot be unambiguously attributed to the bacterial components alone. Additional models are needed to distinguish direct bioactivity from effects driven by antioxidant content, nutritional differences, or microbial metabolism. Furthermore, nematodes have a simpler immune system than humans, lacking adaptive responses and most microbial receptors, and this model is likely insensitive to some of the interactions that could occur in a human [64]. As such, this data should be recapitulated in a mouse model of aging to more fully dissect SBD121’s immunological effects.

In summary, SBD121 is a synbiotic that exhibits multiple complimentary phenotypes that may synergize to modulate pathways associated with inflammaging and healthy aging. By combining antimicrobial activity, production of gut-relevant metabolites, enhancement of epithelial barrier function, reduced chemokine production during inflammatory challenge, and context-dependent modulation of Th17- and Th1-associated cytokine responses, SBD121 may help interrupt the local inflammatory processes that impair healthy aging. Consistent with this model, SBD121 improved lifespan and healthspan in a *C. elegans* aging model, suggesting that these microbial, epithelial, and immune-modulatory phenotypes may translate into organismal benefits. However, the relative contribution of each mechanism remains to be determined. Further in vivo studies will be important to determine whether these mechanisms translate reduced systemic inflammation and enhanced healthspan in mammalian aging.

## 4. Materials and Methods

### 4.1. Microbial Strains

SBD121, a synbiotic composed of lyophilized *Levilactobacillus brevis* strain SBS04254, *Schleiferilactobacillus harbinensis* strain SBS04913, *Lactococcus lactis* strain SBS04916, *Bacillus amyloliquefaciens* strain SBS04877, and prebiotic fibers (oligofructose and organic blueberry powder), has previously been described [3,34]. SBD121 capsule material was resuspended at a concentration of 1.48 × 10^9^ total colony-forming units (CFU)/mL (CFU/mL by strain: 2.11 × 10^8^ CFU/mL *S. harbinensis*; 4.23 × 10^8^ CFU/mL of each: *L. brevis*, *L. lactis*, and *B. amyloliquefaciens*) and ~10.5 mg/mL (capsule to capsule variation: 10% by weight) of each prebiotic component in 1× phosphate-buffered saline (PBS; Catalog (CAT)# BP3991, Thermo Fisher; Waltham, MA, USA) with regular mixing for 25 min at room temperature. When individual SBD121 component strains were required, they were grown in Tryptic Soy Broth (TSB; CAT# 1.00800.0500, Merk KGAG; Darmstadt, Germany) overnight aerobically at 37 °C. *E. coli* (Strain: 1100101; CAT# BAA-2471, American Type Culture Collection (ATCC), Manassas, VA, USA) was grown overnight at 37 °C and washed with 1× PBS. *K. pneumoniae* (SBI00211, Sōlaria Biō Inc., Waltham, MA, USA), *S. aureus* subsp. *aureus* (strain: Rosenbach, CAT# 12600, ATCC, Manassas, VA, USA), and *C. albicans* (Strain: Robin Berkhout; *CAT#* 18804; ATCC, Manassas, VA, USA) were grown overnight aerobically at 37 °C in TSB prior to use. *Porphyromonas gingivalis* Strain: W83 (CAT# BAA-308; ATCC, Manassas, VA, USA), *F. nucleatum* subsp. *nucleatum* Knorr (Strain: VPI 4355 [1612A]; CAT# 25586, ATCC, Manassas, VA, USA), and *S. copri* (strain: CB7 CAT# DSM18205, Leibniz Institute, DSMZ-German Collection of Microorganisms and Cell Cultures GmbH [DSMZ]; Braunschweig, Germany) were grown until turbid (72–96 h) anaerobically at 37 °C prior to use.

### 4.2. Human Cell Culture

Caco-2 (CAT# 86010202, European Collection of Authenticated Cell Cultures, MiliporeSigma; Merck KGAG, Darmstadt, Germany) and HT29 (CAT# 91072201-1VL, European Collection of Authenticated Cell Cultures, MiliporeSigma; Merck KGAG, Darmstadt, Germany) human adenocarcinoma cells were cultured at 37 °C and 5% CO_2_ in Dulbecco’s Modified Eagle Medium (DMEM; CAT# 10566016, Gibco^TM^, Thermo Fisher; Waltham, MA, USA) supplemented with 10% fetal bovine serum (FBS; CAT# 16140071 Gibco^TM^, Thermo Fisher; Waltham, MA, USA), 1× antibiotic–antimycotic (anti-anti; CAT# 15240062, Gibco^TM^, Thermo Fisher; Waltham, MA, USA), and 1× Glutamax^TM^. Cell lines were used from passage 3 to 15.

All human PBMCs were harvested from healthy donors. PBMCs were cultured at 37 °C and 5% CO_2_ in Roswell Park Memorial Institute Medium (RPMI-1640) without phenol red (CAT# 11835030, Gibco^TM^, Thermo Fisher; Waltham, MA, USA) supplemented with 10% FBS, 1× Glutamax^TM^, and 12.5 mM HEPES Buffer (CAT# 15630130, Gibco^TM^, Thermo Fisher; Waltham, MA, USA). PBMCs were maintained in culture for no more than 32 h.

### 4.3. Pathogen Inhibition Assays

SBD121 capsule contents or SBD121 component microbes were inoculated into PYG at a concentration of 1.43 × 10^4^ CFU/mL and grown aerobically at 37 °C for 48 h. SBD121 conditioned supernatants were buffered with 12.5 mM HEPES buffer to a range of 5.0–7.5, centrifuged at 5000 rpm for 10 min, filter-sterilized using 0.2 μm cellulose-acetate filters, and were moved to anaerobic conditions for >20 min to facilitate O_2_ dissipation. Turbid pathogen cultures were inoculated anaerobically into PYG at a dilution of 1:1000. Subsequently, 50 μL of filter-sterilized SBD121, constituent strain conditioned media, or a PYG media control were added to individual 200 μL aliquots of each pathogen to 20% and incubated anaerobically at 37 °C. *S. aureus*, *E. coli*, *K. pneumoniae*, and *C. albicans* were incubated for 24 h. *F. nucleatum* was incubated for 48 h. *P. gingivalis* and *S. copri* were incubated for 72 h. Following incubations, pathogen growth was measured at OD600, and pathogen growth was calculated as a percentage relative to the PYG control growth.

### 4.4. Microbial Metabolite Phenotyping

#### 4.4.1. Neurotransmitter Production Assay

Microbial neurotransmitter production was determined via ELISA (GABA, CAT# EKN45441-96T, Biomatik, Kitchener, ON, Canada; Serotonin, CAT# ADI-900-175, Enzo Life Sciences Inc., Farmingdale, NY, USA). SBD121 capsule contents or SBD121 component microbes were diluted to 2% into 2 mL of brain heart infusion broth (BHI) or BHI supplemented with 0.1% tryptophan for GABA or serotonin production, respectively. Microbes were incubated at 37 °C for 48 h, after which neurotransmitter production was determined per the manufacturer’s protocol.

#### 4.4.2. Indole-Derivative Determination

Microbial production of indole-derived metabolites (indole-3-acetic acid [IAA], indole-3-acetamide, and indole-3-pyruvic acid) were determined via Salkowski assay, as described previously [44]. SBD121 capsule contents or SBD121 component microbes were diluted to 2% in 2 mL of MTGE-anaerobic enrichment broth (CAT# AS-778, Anaerobe Systems, Morgan Hill, CA, USA) supplemented with 0.1% tryptophan. Cultures were grown aerobically at 37 °C for 48 h. After 48 h, supernatants were filtered through 10 kDal size exclusion filters (CAT# UFC801008, MiliporeSigma, Merck KGAG, Darmstadt, Germany) and were mixed at a 1:1 ratio with Salkowski reagent (10 mM iron (III) chloride [CAT#157740-5G, MiliporeSigma, Merck KGAG, Darmstadt, Germany] and 34.3% perchloric acid [CAT#244252-100ML, MiliporeSigma, Merck KGAG, Darmstadt, Germany]) and incubated at 37 °C in the dark for 1 h. Indole-derivative concentrations were determined via 530 nm absorbance, and concentrations were extrapolated from an IAA standard curve (CAT# I3750-25G-A, MiliporeSigma, Merck KGAG, Darmstadt, Germany).

#### 4.4.3. Phenolic Antioxidant Quantification

The ability of SBD121 to produce phenolic antioxidants was determined via a previously developed colorimetric assay [65]. SBD121 capsule contents or SBD121 constituent strains were inoculated into 2 mL of PYG to a concentration of 1.43 × 10^4^ CFU/mL and grown aerobically at 37 °C for 48 h. Subsequently, microbial supernatants were isolated via centrifugation at 5000 rpm for 10 min. A total of 25 μL of each microbial supernatant was diluted into 75 μL of deionized water and 25 μL of 50% Folin–Ciocalteu reagent (CAT# F9252-100ML MiliporeSigma, Merck KGAG, Darmstadt, Germany). Reactions were incubated at room temperature for 6 min, after which 100 μL of 0.708 M Na_2_CO_3_ was added to each reaction. Reactions were incubated at room temperature in the dark for an additional 90 min with shaking. Phenolic antioxidants were quantified as gallic acid equivalents (GAEs) via measuring 765 nm absorbance and concentrations were extrapolated from a gallic acid standard curve (CAT# G7384-100G; MiliporeSigma, Merck KGAG, Darmstadt, Germany).

#### 4.4.4. Microbial Acetate Production

Acetate was quantified as previously described [66]. SBD121 or SBD121 individual microbes were grown anaerobically in BHI at 37 °C for 48 h. Subsequently, acetate concentration from microbial supernatant was determined using a Shimadzu GC-2014 gas chromatograph (Stabilwax-DA GC Capillary Column, 30 m, 0.32 mm ID, 1.00 µm, CAT# 11054; Restek, Bellefonte, PA, USA) with a flame ionization detector.

#### 4.4.5. Microbial L-Lactate Production

SBD121 capsule contents or individual SBD121 component microbes were diluted to 2% into 2 mL of BHI and grown anaerobically at 37 °C for 48 h. Microbial lactate production was determined as per manufacturer’s instructions using the ScienCell^TM^ Research Laboratories L-Lactate assay (CAT# 8308, ScienCell^TM^ Research Laboratories, Carlsbad, CA, USA).

### 4.5. Intestinal Epithelial Cell Polarized Monolayer Preparation

IEC monolayers were established as previously described [66,67]. Briefly, 2.5 × 10^4^ Caco-2 and HT29 IECs were seeded at a ratio of 70:30 onto individual polycarbonate membrane cell culture inserts (6.5 mm diameter, 0.4 µm pore size; 3413, CAT# CLS3396-2EA Costar^TM^; Corning, NY, USA). Each insert contained 200 µL and 1 mL of DMEM in the apical and basolateral chambers, respectively. Monolayers were incubated at 37 °C with 5% CO_2_ for 18 to 21 days. The day before the assay, the inserts were washed with 1× PBS, and the culture medium was replaced with antibiotic-free, phenol red-free MEM, supplemented with 10% FBS and 1× Glutamax^TM^.

The day of the experiment, the trans-epithelial electrical resistance (TEER) of each cell monolayer was determined using a Millicell^TM^ voltohmmeter (Millicell^TM^ ERS-2 with MERSSTX01 electrodes; CAT# MERS00002, EMD Millipore Corporation, Burlington, MA, USA; Electrodes). TEER was measured prior to each experiment (T0) to establish a baseline and 16 h after microbial treatment (T1) to assess TEER change during the experiment (ΔTEER). The values were corrected for background resistance and expressed as Ω × cm^2^.

### 4.6. Barrier Integrity Assays

Polarized IEC monolayers were apically treated with 20 µL (10% *v*/*v*) of either resuspended SBD121 capsule material, a media control (vehicle), or a disruption control (1× PBS-washed *E. coli* diluted in antibiotic-free MEM to an MOI of 20 (2.5 × 10^7^ CFU/mL)). Resuspended SBD121 material was diluted in MEM antibiotic-free medium to a MOI of 25, 5, or 1. Each monolayer was incubated for 16 h at 37 °C with 5% CO_2_. The apical and basolateral supernatants were collected separately for cell viability and cytokine analysis.

### 4.7. Barrier E. coli Challenge Assays

Polarized IEC monolayers were basolaterally treated with a media control or MOI 20 *E. coli* diluted in antibiotic-free MEM for 4 h at 37 °C with 5% CO_2_. Subsequently, basolateral chambers were supplemented with polymyxin B (CAT# P1189-1, MiliporeSigma, Merck KGAG, Darmstadt, Germany), a Gram-negative specific antibiotic, and each monolayer was apically treated with 20 µL (10% *v*/*v*) of a media control (vehicle) or SBD121 capsule material resuspended to an MOI of 5. Each monolayer was incubated for an additional 16 h at 37 °C with 5% CO_2_.

### 4.8. IEC Cytokine Challenge Assays

HT29 challenge assays were performed as previously described [68]. A total of 500 µL of HT29 cells were seeded into 48 well plates at a concentration of 1 × 10^5^ cells per well. Cells were incubated at 37 °C with 5% CO_2_ for 14 days to promote differentiation into unpolarized IEC monolayers. On the day of the experiment, cells were washed with 1× PBS and media was replaced with 450 µL antibiotic-free MEM. Cells were then treated with 5 ng/mL TNF-α (CAT# PHC3016, Thermo Fisher, Waltham, MA, USA) or a media control. Concurrently, each well received 50 µL (10% *v*/*v*) of a media control (vehicle), 100 ng/mL of LPS (CAT# tlr-eblps, Invivogen; San Diego, CA, USA), or SBD121 at a concentration of MOI 25, 5, 1, or 0.2. Cells were then incubated for 24 h at 37 °C with 5% CO_2_. Cell viability was determined via lactate dehydrogenase activity per manufacturer’s instructions (CAT# G1780, Promega; Fitchburg, WI, USA). Cytokine responses were determined via ELISA performed as per manufacturer’s instructions (BioLegend, San Diego, CA, USA: IL-8/CXCL8 [CAT# 431501]; and R&D systems, Minneapolis, MN, USA: CXCL1 [CAT# DY275]).

### 4.9. PBMC Isolation

Human PBMCs were isolated using polymorphprep (CAT# NC0863559, Fisher Scientific, Waltham, MA, USA) [69]. Whole human blood was harvested from healthy donors (females, *n* = 3, age 33–41, average age: 36 yrs; males, *n* = 4, age 34–37, average age: 35.5 yrs) and was layered over an equal volume of Polymorphprep and centrifuged at 550× *g* for 35 min with no deceleration. Blood cells were separated into two bands, an upper band containing isolated PBMCs and a lower band containing isolated neutrophils. The upper band was removed via syringe and centrifuged at 500× *g* for 5 min. The resulting pellet was resuspended in 1 mL of RPMI-1640 and 3 mL of red blood cell (RBC) lysis buffer (CAT#11814389001, MiliporeSigma, Merck KGAG, Darmstadt, Germany). Cells were incubated for 10 min after which 6 mL of RPMI-1640 was added to each suspension and centrifuged at 500× *g* for 5 min. Resulting RBC-free pellets were resuspended in RPMI-1640 to a concentration of 6.67 × 10^5^ cells/mL. Donor information may be found in Table 1. Each donor was assessed in 3–5 technical replicates; donor means were used for statistical analysis.

### 4.10. SBD121 Treatment of PBMCs

In 1.5 mL, one million cells were treated with media alone (unchallenged control) or 100 ng/mL of LPS (inflammatory challenge) for 30 min at 37 °C with 5% CO_2_ [70,71]. Subsequently cells were washed with 1× PBS and resuspended in antibiotic-free RPMI-1640. Cells were then treated with resuspended SBD121 capsule contents diluted in RPMI to an MOI of 2, 0.4, or 0.08 [71]. Cells were incubated for 16 h at 37 °C with 5% CO_2_, after which supernatants were harvested for viability and cytokine response analysis while cells were resuspended in DNA/RNA Safe (CAT# R1100-50, Zymo Research, Irvine, CA, USA) and stored at −80 °C for qRT-PCR analysis. Cell viability was determined via lactate dehydrogenase activity as described above. Cytokine responses were determined, as per manufacturer’s instructions, via ELISA (Thermo Fisher, Waltham, MA, USA: TNF-α (CAT# 88-7346-88), IL-23p19 (CAT# 88-7237-88); BioLegend, San Diego, CA, USA: IL-12p70 (CAT# 431701), IL-6 (CAT# 430501), IL-1β (CAT# 437016), IL-8 (CAT# 431501), CCL2 (CAT# 38804), CCL20 (CAT# 441404); and R&D systems, Minneapolis, MN, USA: IFN-γ (CAT# DY285B), CXCL1 (CAT# DY275), IL-1RA (CAT# DY280-05), IL-10 (CAT# DY217B)).

Total PBMC RNA was isolated using the Quick-RNA^TM^ miniprep plus kit (CAT# R1058; Zymo Research, Irvine, CA) and converted to cDNA using the iScript^TM^ gDNA Clear cDNA Synthesis Kit (CAT# 1725034, Bio-Rad, Hercules, CA, USA) per manufacturer’s instructions. cDNA was examined by real-time PCR to determine the absolute gene expression of *CDKN1A* (p21), *CDKN2A* (p16), and *GAPDH* on a Biorad CFX96 Touch Real-Time PCR detection system using the PrimeTime^TM^ gene expression master mix (CAT# 1055770, Integrated DNA Technologies (IDT), Coralville, IA, USA) and commercially available primer–probe sets (IDT, Coralville, IA. *CDKN1A*: Hs.PT.58.40874346.g; *CDKN2A*: Hs.PT.58.40743463.g; *GAPDH*: Hs.PT.39a.22214836). Primer sequences may be found in Table 2. Absolute gene expression was extrapolated from standard curves and data was presented as copies of the target transcript per copies of *GAPDH* transcript.

### 4.11. C. elegans Lifespan and Activity Assay

All procedures were performed by NemaLife Inc. using their propriety methods. *C. elegans* were raised on standard agar plates at 20 °C seeded with live *E. coli* OP50 from hatching until the L4 stage. At the L4 stage, the worms were loaded into NemaLife chips at a density of ~60 animals per chip (*n* = 2–6 chips per experimental group). The worms were then switched to a diet of 25 mg/mL live OP50 resuspended in liquid NGM. The animals in each chip were imaged to record the population size and baseline activity levels after 24 h. Chips were then washed with NGM buffer for 90 s to remove waste products and any progeny laid in the previous 24 h. After washing the chips, the media was exchanged with media for the appropriate experimental condition. Experimental conditions included SBD121 at a concentration of 5 × 10^9^ CFU/mL, suspended in a prebiotic containing buffer that corresponds to the prebiotic components of SBD121. A no-treatment negative control was performed using *E. coli* OP50 as the food source (25 mg/mL OP50). A positive control was also performed by administering resveratrol (100 μM with 0.1% dimethyl sulfoxide) diluted with liquid NGM to worms fed *E. coli* OP50 (25 mg/mL) as the food source. The media on all chips were exchanged every 24 h to remove waste and any progeny. On days 8, 12, 16, and 18 of animal life, videos were recorded of the animals remaining on the chip to quantify their survival rate and locomotor activity levels.

Locomotory activity ‘a’ of the worms was determined by NemaLife’s Movement Tracker software [72]. The software applies a bounding box to each detected animal, and the pixel intensity correlation is calculated between 30 s separated frames. If the normalized pixel intensity correlation between the two bounding boxes is unity, then a = 0, indicating that the pixel intensities associated with the detected worm objects are fully correlated, and the animal is stationary in this time interval. Alternatively, if there is no correlation, then a = 1, indicating that the animal has moved out of its bounding box. Using this approach, three activity scores were obtained per animal from a single video. These activity scores were then grouped by condition and replicate to visualize differences between experimental cohorts.

### 4.12. Statistical Analysis

Data were analyzed with the Prism 11 graphing and analysis software (GraphPad Software, Boston, MA, USA). Normality was determined by Shapiro–Wilk test, and for unnormalized data variance was determined by Brown–Forsythe test. When assumptions were met, one-way ANOVA with Tukey’s HSD, to correct for multiple comparisons, was used. One-way ANOVA with Dunn’s multiple comparisons test was used to correct for multiple comparisons, when all comparisons were made against a control condition. When distribution assumptions were not met, the Kruskal–Wallis test with Dunn’s multiple comparisons test was used. When assumptions of standard deviation normality were not met, Welch ANOVA with Dunnet’s T3 multiple comparison test was used. Mixed-effects analysis with Geisser–Greenhouse correction and Dunnett’s multiple comparison test was used to determine significance for nematode survival studies. When normalized data was analyzed by challenge state, as in Figure 3, Figure 4 and Figure 5 and Figures S2–S4, individual statistical analysis was performed for each challenge state (challenge-naïve or LPS challenge). For PBMC sex-stratified analyses, donor means were used as the unit of analysis. Given the limited sample size (*n* = 3 female, *n* = 4 male donors), these comparisons are considered preliminary and hypothesis-generating.

## 5. Conclusions

In conclusion, SBD121 demonstrates complementary antimicrobial, metabolic, epithelial, and immune-modulatory activities that may converge to modulate inflammatory pathways relevant to inflammaging and support healthy aging. Importantly, SBD121 reduced inflammatory chemokine signaling and immunosenescence-associated markers without evidence of broad immunosuppression, instead suggesting context-dependent immune modulation that includes reduced Th17-associated responses and preservation or enhancement of Th1-associated responses. Together with improvements in lifespan and healthspan, these data provide a mechanistic rationale for future investigation in mammalian aging models, which will be necessary to determine whether these effects translate to health benefits during aging.

## 6. Patents

Issued patents relating to data within this work: US12,016,891 and US 12,440,523.

## Figures and Tables

**Table 1:** PBMC donor demographics.

Donor Number	Icon	Sex	Age (yrs)
1	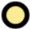	Female	33
2	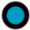	Female	34
3	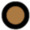	Female	41
4	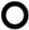	Male	37
5	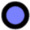	Male	37
6	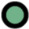	Male	34
7	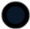	Male	34

**Table 2:** PCR primers and probes used in this study.

IDT Catalog Number	Target	Sequence
Hs.PT.58.40874346.g	Primer 1 *CDKN1A*	GAGACTAAGGCAGAAGATGTAGAG
Hs.PT.58.40874346.g	Primer 2 *CDKN1A*	GCAGACCAGCATGACAGAT
Hs.PT.58.40874346.g	Probe *CDKN1A*	/5Cy55/TTCCTCTTGGAGAAGATCAGCCGG/3BHQ-3/
Hs.PT.58.40743463.g	Primer 1 *CDKN2A*	TGAGCTTTGGTTCTGCCATT
Hs.PT.58.40743463.g	Primer 2 *CDKN2A*	AGCTGTCGACTTCATGACAAG
Hs.PT.58.40743463.g	Probe *CDKN2A*	/56-FAM/TAGCAGTGT/ZEN/GACTCAAGAGAAGCCAGT/3IABkFQ/
Hs.PT.39a.22214836	Primer 1 *GAPDH*	TGTAGTTGAGGTCAATGAAGGG
Hs.PT.39a.22214836	Primer 2 *GAPDH*	ACATCGCTCAGACACCATG
Hs.PT.39a.22214836	Probe *GAPDH*	/5TexRd-XN/AAGGTCGGAGTCAACGGATTTGGTC/3BHQ_2/

## Data Availability

The data presented in this study are available on request from the corresponding author.

## Supplementary Materials

The following supporting information can be downloaded at https://www.mdpi.com/article/10.3390/ijms27146369/s1.

## Abbreviations

The following abbreviations are used in this manuscript:

Anti-anti	Antibiotic–antimycotic
ATCC	American Type Culture Collection
BHI	Brain heart infusion broth
CAT	Catalog
CCL	C-C motif chemokine ligand
CDKN	Cyclin-dependent kinase inhibitor
CFU	Colony-forming unit
CXCL	C-X-C motif chemokine ligand
DMEM	Dulbecco’s Modified Eagle Medium
ELISA	Enzyme-linked immunosorbent assay
FBS	Fetal bovine serum
GAE	Gallic acid equivalents
GABA	Gamma-aminobutyric acid
GI	Gastrointestinal
GM-CSF	Granulocyte-macrophage colony-stimulating factor
IAA	Indole-3-acetic acid
IAAE	Indole-3-acetic acid equivalents
IEC	Intestinal epithelial cell
IFN-γ	Interferon gamma
IL	Interleukin
IL-1RA	Interleukin-1 receptor antagonist
LPS	Lipopolysaccharide
MEM	Minimum essential medium
MOI	Multiplicity of interaction
NGM	Nematode growth medium
PBMC	Peripheral blood mononuclear cell
PBS	Phosphate-buffered saline
PYG	Peptone yeast extract broth with glucose
qRT-PCR	Quantitative reverse transcriptase polymerase chain reaction
RPMI-1640	Roswell Park Memorial Institute Medium
SASP	Senescence-associated secretory pattern
TEER	Transepithelial electrical resistance
Th	T helper
TSB	Tryptic soy broth
TNF-α	Tumor necrosis factor alpha
ΔTEER	Change in TEER
