# Supplementary material for: A Defined Synbiotic Produces Immunomodulatory Metabolites, Engages Gut–Immune Pathways Relevant to Inflammaging, and Supports Healthy Aging in a Nematode Model
Source: Int J Mol Sci. 2026 Jul 17;27(14):6369. doi: 10.3390/ijms27146369 (PMC13409942; doi:10.3390/ijms27146369)
Supplement: Supplementary file 1 [file ijms-27-06369-s001.zip › ijms-4373377-supplementary.pdf]

Growth (% untreated pathogen)	*L. brevis*		*S. harbinensis*		*L. lactis*		*B. amyloliquefaciens*		*Synbiotic*	
	SBS04254		SBS04913		SBS04916		SBS04877		SBD121	
	Average	*Stdev*	Average	*Stdev*	Average	*Stdev*	Average	*Stdev*	Average	*Stdev*
K. pneumonaie	94	*5*	103	*2*	85 *	*10*	1 ****	*1*	1****	*1*
P. gingivalis	148	*105*	188	*194*	146	*143*	4	*5*	1	*2*
E. coli	93	*3*	103	*9*	89	*5*	1 ****	*1*	9 ****	*13*
S. copri	88	*27*	66	*5*	54	*13*	60	*39*	19**	*15*
C. albicans	106	*12*	155	*73*	142	*65*	77	*45*	50	*10*
F. nucleatum	102	*5*	105	*2*	93	*21*	58**	*16*	68 *	*0*
S. aureus	97	*25*	95	*3*	77	*11*	75	*49*	56	*28*

Table S1. SBD121 inhibits pathogen growth. SBD121 or its constituent strains were grown aerobically for 48 h. After which the bacteria-free conditioned supernatants or a medium control were applied to potential pathogenic bacteria (*Klebsiella pneumoniae, Porphyromonas gingivalis, Escherichia coli, Segatella copri, Candida albicans, Fusobacterium nucleatum,* and *Staphylococcus aureus*). Data represent the mean of 2 -3 independent experiments, each with 3-6 technical replicates per experiment. Significance was determined via One-way ANOVA with Dunnet's HSD, comparing all conditions to SBD121 the media background.

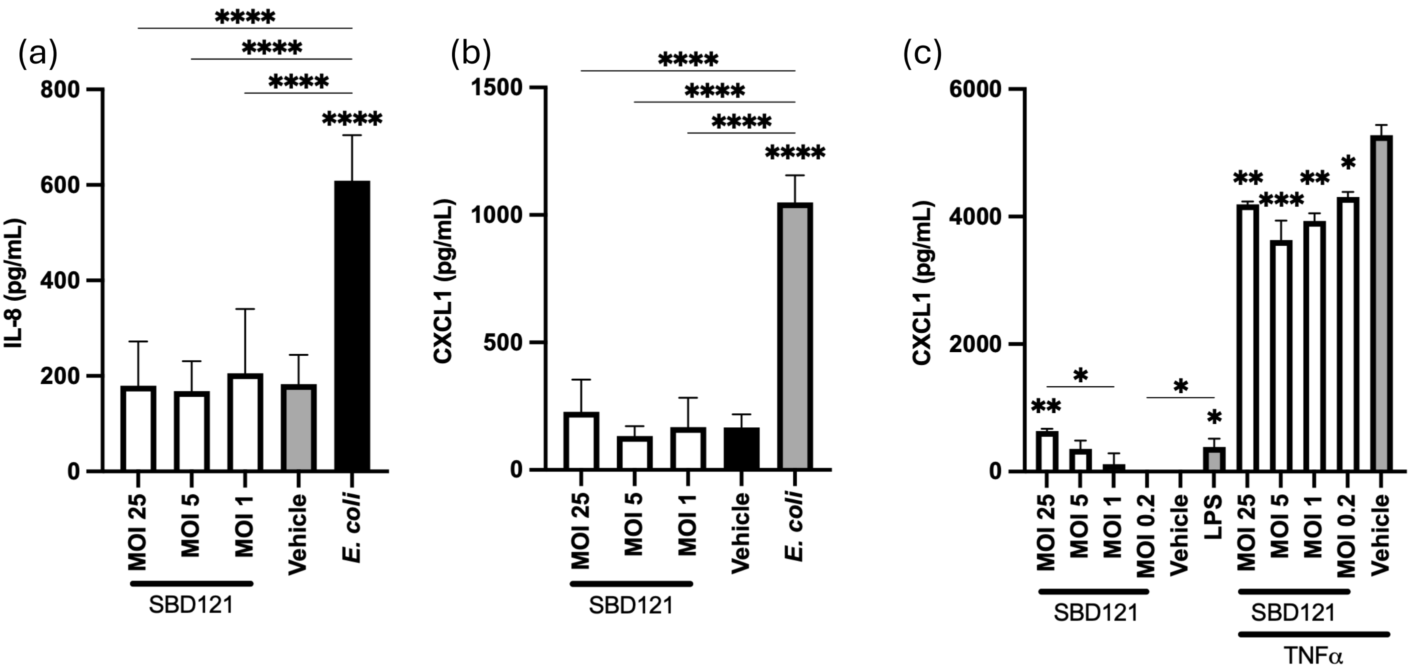

Figure S1. SBD121 does not induce chemokine secretion in polarized IEC models and reduces CXCL1 secretion by non-polarized IECs during inflammatory challenge. (a and b) Mature, polarized IEC monolayers (Caco-2 and HT29 cells) were treated with a media control (vehicle), an *E. coli* disruption control, or SBD121 capsule contents at MOIs 25, 5 or 1). After 16 hrs cytokine responses were determined via ELISA [**(a)** IL-8 and **(b)** CXCL1]. These data are from a representative of three experiments. **(c)** Non-polarized HT29 monolayers were co-treated with TNF-*α* and SBD121 (MOI 25, 5, 1, or 0.2) or a vehicle control, or a stimulatory control (LPS). After 24 h supernatants were harvested and IL-8 concentrations were determined via ELISA. Data presented is from the experiment presented in Figure 1c and is representative of three experiments. Bars indicate the mean of the condition ± the standard deviation (SD). Significance was determined by one-way ANOVA with Tukey's HSD. Asterisks lacking comparison bars identify significance relative to the vehicle control. * = p <0.05, ** = p <0.01, **** = p <0.0001

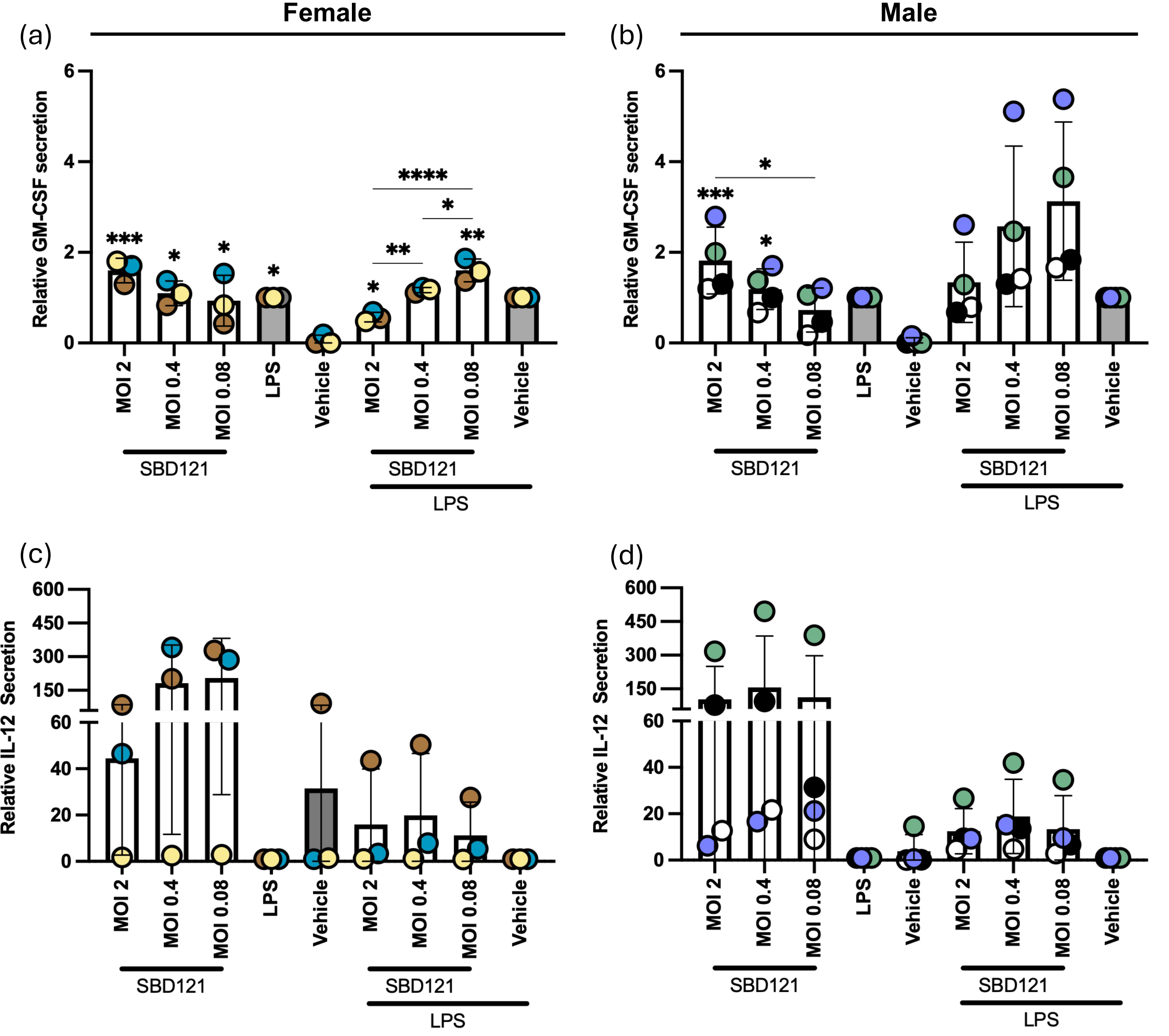

Figure S2. SBD121 administration alters PBMC secretion of Th17- and Th1-associated cytokines at baseline and after inflammatory challenge. Healthy donor PBMCs (three female **[a, c, and e]** and four male **[b, d, and f]**) were pretreated with media or LPS (100 ng/mL), inducing an inflammatory response. After which cells received SBD121 capsule contents (MOI 2, 0.4, or 0.08), a stimulatory control (LPS) or a media control (Vehicle). T cell polarizing cytokine secretion was determined via ELISA after 16 h (GM-CSF **[a and b]** and IL-12 **[c and d]**). Multiple donors were compared by normalizing inflammation naïve and LPS challenge conditions to LPS and LPS-challenged, vehicle-treated controls, respectively. Columns indicate the mean ± SD for all the donors of an individual sex. Points indicate individual donors and are color coded by donor. Females: Donor 1 ; Donor 2 ; Donor 3 . Males: Donor 4; Donor 5; Donor 6; Donor 7). Significance was determined by One-way ANOVA with Tukey's HSD. Asterisks without comparison bars indicate significance relative to the vehicle control. * = $p < 0.05$, ** = $p < 0.01$, *** = $p < 0.001$, **** = $p < 0.0001$

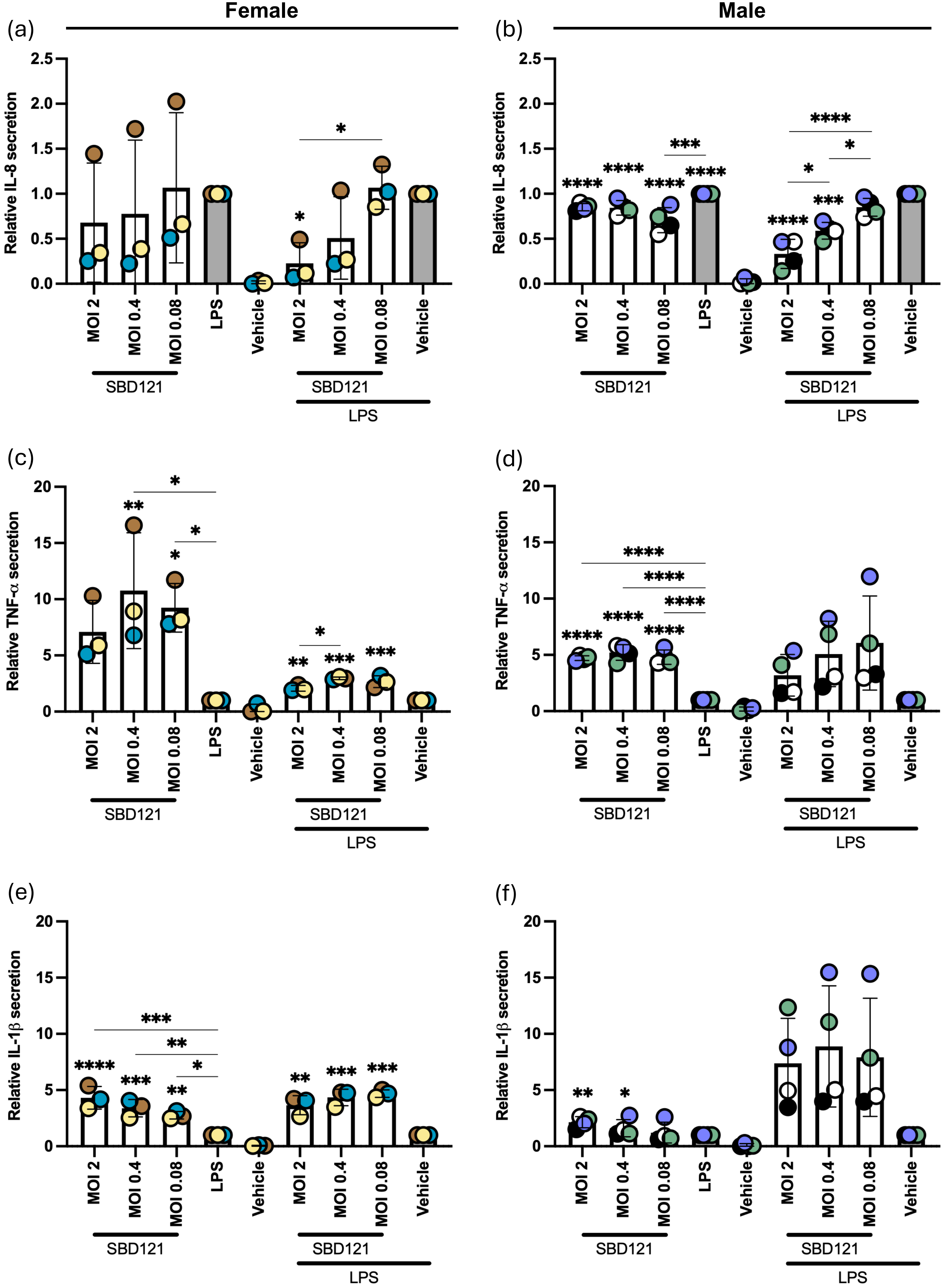

Female
Male
(a)
(b)
(c)
(d)
(e)
(f)
Relative IL-8 secretion
Relative TNF-α secretion
Relative IL-1β secretion
MOI 2
MOI 0.4
MOI 0.08
LPS
Vehicle
SBD121
LPS

Figure S3. SBD121 modulates PBMC secretion of innate immunity cytokines at basal and after inflammatory challenge. PBMCs from seven healthy donors (three female **[a, c, and e]** and four male **[b, d, and f]**) were pretreated with media or an inflammatory challenge (100 ng/mL LPS). After which cells were exposed to SBD121 capsule contents (MOI 2, 0.4, or 0.08), a stimulatory control (LPS) or a media control (Vehicle). Innate immunity cytokine secretion was determined via ELISA after 16 h (IL-8 **[a and b]**, TNF-α **[c and d]**, and IL-1β **[e and f]**). Donors were compared by normalizing inflammation naïve to the LPS control ± standard deviation for the donors of an individual condition. Points indicate individual donors and are color coded and LPS challenge conditions to the LPS-challenged, vehicle-treated controls, respectively. Columns indicate the mean by donor. Females: Donor 1 ; Donor 2 ; Donor 3 . Males: Donor 4 ; Donor 5 ; Donor 6; Donor 7). Significance was determined by One-way ANOVA with Tukey's HSD. Asterisks without comparison bars indicate significance relative to the vehicle control. * = $p < 0.05$, ** = $p < 0.01$, *** = $p < 0.001$, **** = $p < 0.0001$

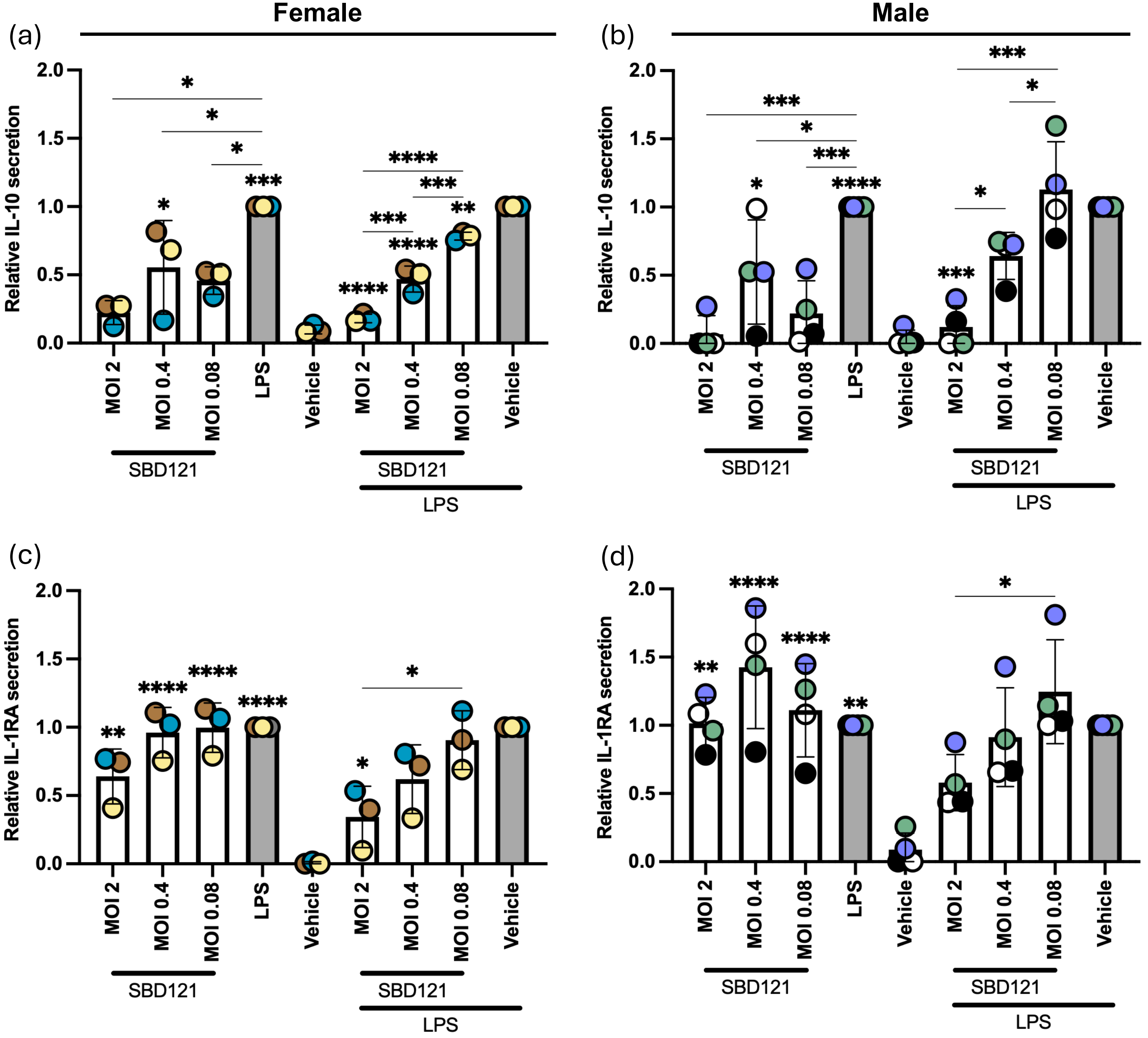

Figure S4. SBD121 treatment dose-dependently induces and reduces anti-inflammatory cytokine secretion under baseline and inflammatory conditions, respectively. PBMCs from three healthy female **(a and c)** and four healthy male **(b, d, and f)** were exposed to media or an inflammatory challenge (LPS, 100 ng/mL). Subsequently, cells received SBD121 capsule contents (MOI 2, 0.4, or 0.08), a stimulatory control (LPS) or a media control (Vehicle). Anti-inflammatory cytokine secretion was examined via ELISA after 16 h (IL-10 **[a and b]** and IL-1RA **[c and d]**). Multiple donors were compared by normalizing inflammation naïve and LPS challenge conditions to LPS and LPS-challenged, vehicle-treated controls, respectively. Columns represent the mean ± SD for all the donors of an individual sex. Points indicate individual donors and are color coded by donor. Females: Donor 1 ; Donor 2 ; Donor 3 . Males: Donor 4; Donor 5; Donor 6; Donor 7). Significance was determined by One-way ANOVA with Tukey's HSD. Asterisks without comparison bars indicate significance relative to the vehicle control. * = $p < 0.05$, ** = $p < 0.01$, *** = $p < 0.001$, **** = $p < 0.0001$

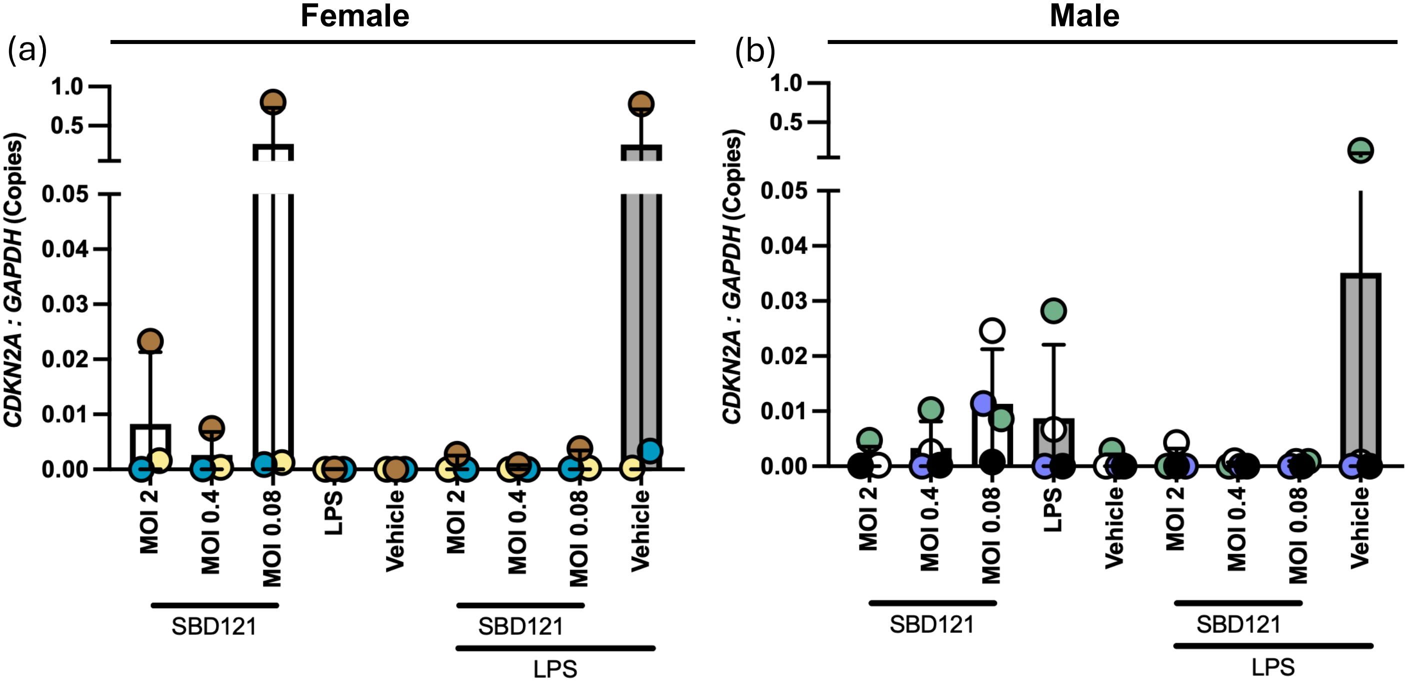

Figure S5. SBD121 administration did not significantly impact PBMC p16 expression. PBMCs from healthy donors (three female **[a]** and four male **[b]**) were exposed to media or LPS (100 ng/mL), to produce an inflammatory response. After which cells received SBD121 capsule contents (MOI 2, 0.4, or 0.08), a stimulatory control (LPS) or a media control (Vehicle) for 16 h. *CDKN2A* (p16) gene expression was determined by qRT-PCR. Copy numbers were extrapolated using a standard curve, and data is presented as the copies of *CDKN2A* per copy of *GAPDH*. Columns indicate the mean ± standard deviation for all the donors of an individual sex. Points indicate individual donors and are color coded by donor. Females: Donor 1 ; Donor 2 ; Donor 3 . Males: Donor 4; Donor 5; Donor 6; Donor 7). Significance was determined by One-way ANOVA with Tukey's HSD. Asterisks without comparison bars indicate significance relative to the vehicle control. * = $p < 0.05$, ** = $p < 0.01$, *** = $p < 0.001$, **** = $p < 0.0001$
